# Supplementary material for: Public Health Implications of Introducing Human Papillomavirus (HPV) Vaccination in Pakistan: A protocol for a mixed-method study to explore community perceptions and health system preparedness
Source: PLoS One. 2025 May 22;20(5):e0323826. doi: 10.1371/journal.pone.0323826 (PMC12097576; doi:10.1371/journal.pone.0323826)
Supplement: Attachment — Submitted filename: Response to reviewer 2 review.docx [file pone.0323826.s003.docx]

Dear Editor,

We are thankful to the worthy reviewers for sparing their valuable time to review our manuscript. Please find the description and justification of point-to-point comments and line

| **S.#** | **Comment** | **Response** | **Reply/Changes** |
| --- | --- | --- | --- |
| 1 | The English needs further editing. Many definite and indefinite articles are missing in the text.Avoid mixing both American and British English. Stick to one. | Document is thoroughly reviewed and edited | Throughout document |
| 2 | The Aim is to look at factors influencing the uptake of HPV vaccine, not HPV. Correct this. | Corrected | Page 6  Line 186-187 |
| 3 | Objective 2: be specific about the country (Pakistan in this case | Added accordingly | Page 6  Line 190-191 |
| 4 | Objective 3: be specific about the community | Specific community setting added in objective and methodology both | Page 6 Line 192-193  Page 7 Line 225-226 |
| 5 | Under HPV Vaccination belief subheading, HBM should be written in full at first use. | Added in full form | Page 8  Line 271 |
| 6 | Qualitative data: This data was to be collected till data saturation is achieved. However, the authors stated that only 4 FGDs will be conducted with the KIIs. Please, reconcile these to clearly show how sample size will be determined. | Sample size for qualitative data is determined by data saturation , a point where new information no longer contributes to understanding of phenomena of interest, our initial sample size are based on normal saturation points in comparable research^30^. Since point of saturation cannot be predicted in advance so we planed to recruit minimum of 25-30 caregivers and potential recipients , 8-10 policy makers and 25-30 healthcare providers which is in accordance with the typical points of data saturation reported in qualitative studies^31^. However we are willing to modify our sample size if needed, and we will continue to recruit until saturation is reached to ensure a indepth knowledge of the factors influencing HPV vaccine uptake. | Page 11  Line 370-378 |
